# The use of ICU resources in CAR-T cell recipients: a hospital-wide study

**DOI:** 10.1186/s13613-022-01036-2

**Published:** 2022-08-17

**Authors:** Sandrine Valade, Michael Darmon, Lara Zafrani, Eric Mariotte, Virginie Lemiale, Swann Bredin, Guillaume Dumas, Nicolas Boissel, Florence Rabian, André Baruchel, Isabelle Madelaine, Jérôme Larghero, Anne Brignier, Etienne Lengliné, Stéphanie Harel, Bertrand Arnulf, Roberta Di Blasi, Catherine Thieblemont, Elie Azoulay

**Affiliations:** 1 AP-HP, Hôpital Saint-Louis, Medical ICU, 1 avenue Claude Vellefaux, 75010 Paris, France; 2 Université de Paris, Paris, France; 3 AP-HP, Hôpital Saint-Louis, Hematology Adolescent and Young Adults Unit, URP-3518 Paris, France; 4 Department of Pediatric Hematology, AP-HP, Robert Debré Hospital, Paris, France; 5 Department of Pharmacy, AP-HP, Hôpital Saint Louis, Paris, France; 6 Cell Therapy Unit, AP-HP, Université de ParisHôpital Saint-Louis, Paris, France; 7 Clinical Investigation Center in Biotherapies (CBT-501), INSERM, Paris, France; 8 AP-HP, Saint Louis Hospital, Therapeutic Apheresis Unit, Paris, France; 9 AP-HP, Hôpital Saint Louis, Hematology department, Paris, France; 10 Immuno-Hematology Department, AP-HP, Hôpital Saint LouisSaint-Louis Hospital, Paris, France; 11 Hemato-oncology, DMU HI, AP-HP, Hôpital Saint-Louis, Research Unit NF-kappaB, Différenciation et Cancer, 75010 Paris, France

**Keywords:** Anti-CD19 chimeric antigen receptor, Intensive care, Hematological malignancies, Sepsis, Performance status

## Abstract

**Background:**

CAR-T cell (chimeric antigen receptor T) therapy has emerged as an effective treatment of refractory hematological malignancies. Intensive care management is intrinsic to CAR-T cell therapy. We aim to describe and to assess outcomes in critically ill CAR-T cell recipients.

**Study design and methods:**

Hospital-wide retrospective study. Consecutive CAR-T cell recipients requiring ICU admission from July 2017 and December 2020 were included.

**Results:**

71 patients (median age 60 years [37–68]) were admitted to the ICU 6 days [4–7] after CAR-T cell infusion. Underlying malignancies included diffuse large B cell lymphoma (*n* = 53, 75%), acute lymphoblastic leukemia (17 patients, 24%) and multiple myeloma (*n* = 1, 1.45%). Performance status (PS) was 1 [1–2]. Shock was the main reason for ICU admission (*n* = 40, 48%). Isolated cytokine release syndrome (CRS) was the most common complication (*n* = 33, 46%), while 21 patients (30%) had microbiologically documented bacterial infection (chiefly catheter-related infection). Immune effector cell-associated neurotoxicity syndrome was reported in 26 (37%) patients. At ICU admission, vasopressors were required in 18 patients (25%) and invasive mechanical ventilation in two. Overall, 49 (69%) and 40 patients (56%) received tocilizumab or steroids, respectively.

Determinant of mortality were the reason for ICU admission (disease progression vs. sepsis or CRS (HR 4.02 [95%CI 1.10–14.65]), Performance status (HR 1.97/point [95%CI 1.14–3.41]) and SOFA score (HR 1.16/point [95%CI 1.01–1.33]).

**Conclusions:**

Meaningful survival could be achieved in up to half the CAR-T cell recipients. The severity of organ dysfunction is a major determinant of death, especially in patients with altered performance status or disease progression.

**Supplementary Information:**

The online version contains supplementary material available at 10.1186/s13613-022-01036-2.

## Background

CAR-T cell (chimeric antigen receptor T) therapy has emerged as an effective treatment in relapsed/refractory B-cell hematological malignancies (especially in acute lymphoblastic leukemia, ALL; diffuse large B cell lymphoma, DLBCL). This innovative T-cell immunotherapy is based on the genetic modification of autologous cytotoxic T lymphocytes. The specific recognition of a tumoral antigen through the CAR, results in tumor lysis [1]. In the pivotal clinical trials in patients with relapsed and refractory aggressive B-cell lymphoma (JULIET, ZUMA 1, TRANSCEND), the best complete response rate ranges between 39 and 54% [2–4]. Overall survival at 1 year is described between 40 and 45%. In pediatric and young adult patients with acute lymphoblastic leukemia (ELIANA phase 2 trial), an overall response rate of 81% was achieved at 3 months in 75 patients who received tisagenlecleucel and overall survival at 1 year was 76% [5]. These results hold strong promise in the absence of an available alternative treatment. Nevertheless, CAR-T cell-related complications can be life threatening in patients often frail and deeply immunocompromised, leading them to the intensive care unit (ICU) [6, 7].

Cytokine released syndrome (CRS) is the most commonly observed complication following CAR-T cell infusion, with an incidence reported between 58 and 93%, depending on the underlying hematological malignancy, the type of CAR and the tumor burden [3, 5, 8, 9]. It occurs in a median time of 2 to 3 days after CAR-T cell infusion [2, 3, 5, 8, 10]. Pathophysiology is based on the activation and expansion of CAR-T cells in vivo, leading to macrophages recruitment and the secretion of pro-inflammatory cytokines [9, 11, 12]. CRS can be severe, resulting in serious organ failures. Specific treatment relies on a recombinant humanized monoclonal antibody that acts as an interleukin 6 (IL-6) receptor antagonist (tocilizumab). Steroids are administered in the absence of response and in the sickest patients [10, 13–15].

Immune effector Cell-Associated Neurotoxicity Syndrome (ICANS) [16] represents the second specific complication of CAR-T cell therapy, occurring in a median time of 4 to 10 days after CAR-T cell infusion. It involves 20 to 60% of patients [2, 3, 5, 17–20] and rarely occurs in the absence of CRS [11, 16, 19, 21]. Its pathophysiology is complex and several mechanisms are combined (i) a disruption of the blood–brain barrier with an increase in pro-inflammatory cytokines in the cerebrospinal fluid and cerebral parenchyma, (ii) an endothelial activation leading to capillary hyperpermeability, and (iii) a glial activation as well as an increase in excitatory neurotransmitters [18, 19, 22, 23]. Clinical symptoms are extremely diverse and specific therapeutic management relies on steroids administration [10, 14] or an anti-IL1 in refractory cases [15, 24, 25]. Administration of tocilizumab is not an option, as it may worsen neurotoxicity by increasing IL-6 levels in cerebrospinal fluid [10, 18, 19].

These severely immunocompromised patients may also develop unspecific complications, such as sepsis [26], that also require intensive care management [6, 7].

Only few studies specifically focused on the management of critically ill CAR-T cell recipients [27]. We sought to assess outcomes in these patients.

## Methods

### Patients and methods

**Fig. 1:**
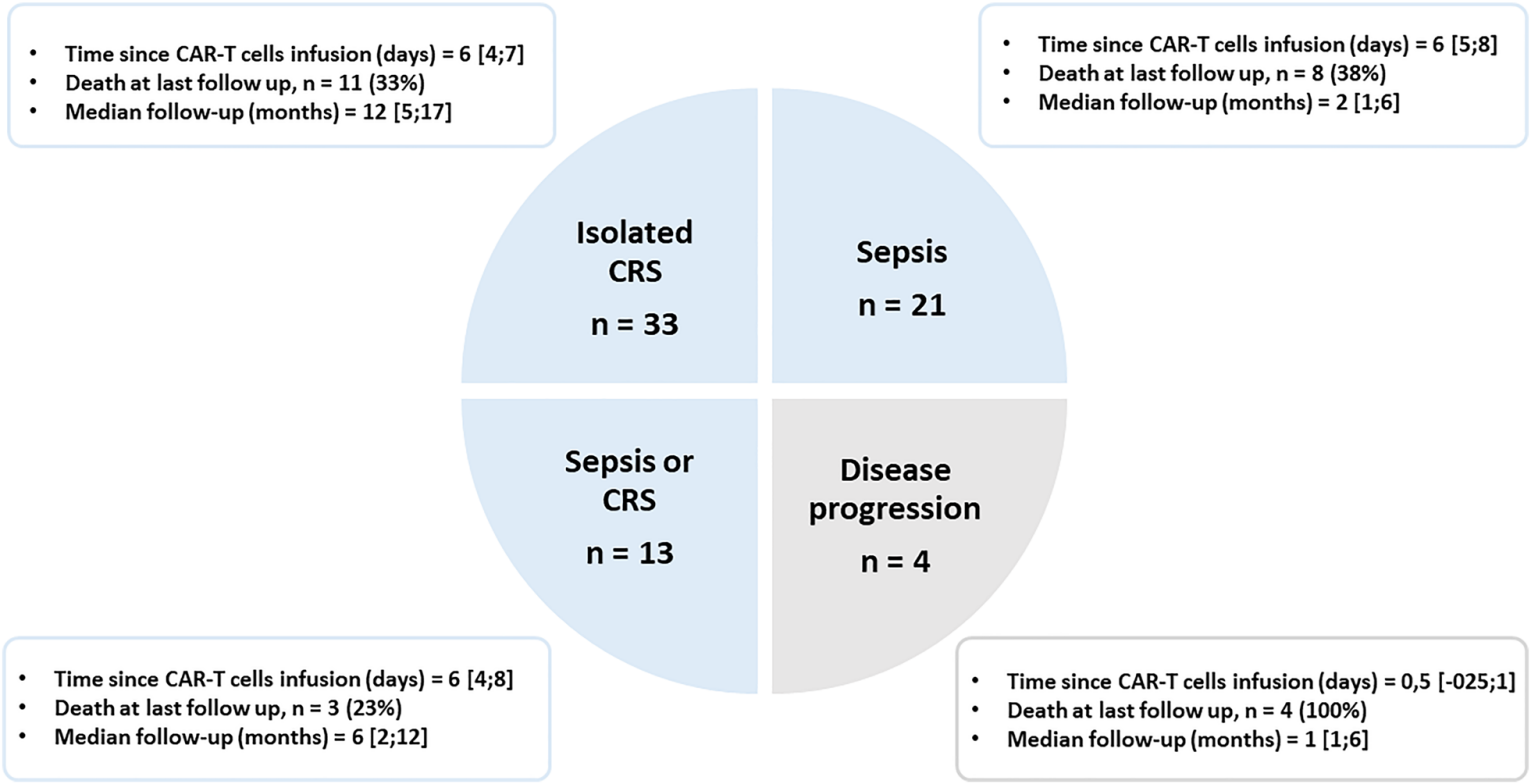
Characteristics of patients according to the reason for ICU admission. *CRS* cytokine release syndrome

This is a hospital-wide retrospective study conducted between July 2017 and December 2020 in the Saint Louis hospital (Paris, France). Consecutive CAR-T cell recipients who required ICU admission within 30 days of CAR infusion were included. Of note, after CAR-T cell infusion, no patient was denied for ICU admission, regardless of organ failure. In line with previous studies performed in our center regarding influence of delayed ICU admission on outcome of critically ill immunocompromised patients [28], ICU admission was considered independently to severity in following situations: sepsis with any degree of severity when fluid bolus is required; acute respiratory failure with oxygen support above or equal to 3L/min; acute kidney injury; any new organ dysfunction or situation at high risk of organ dysfunction and requiring close monitoring. In case patients had been readmitted, only the first admission was considered. Patients were categorized into four groups that were defined preliminary to the study: “sepsis” (patients with a microbiologically documented bacterial infection), “CRS” (patients meeting CRS definition and without clinical or microbiologically documented infection), “disease progression” and “sepsis or CRS” (patients meeting CRS definition, without microbiologically documented infection but with clinical or radiological presentation which may evoke sepsis). In this last group, patients may experience fever, hypotension or hypoxemia as a consequence of sepsis, CRS or any degree of both mechanisms (Fig. 1).

CRS grading was based on the ASBMT classification [16] (Additional file 1: Table S1) and immune effector cell-associated neurotoxicity syndrome (ICANS) was evaluated using the CAR-T cell therapy-associated TOXicity score (CARTOX) (Additional file 1: Table S2) [12]. Organ toxicities were defined following the Common Terminology Criteria for Adverse Events (CTCAE) [29]. ICU specialists followed guidelines related to the management of CRS and ICANS [11]. The Sepsis-Related Organ Failure Assessment (SOFA) score was calculated at ICU admission to assess organ failures [30]. Performance status was measured according to the Eastern Cooperative Oncology Group (ECOG) score [31]. Neutropenia was defined by a white blood cells count below 1000 cells/mm^3^.

This study was approved by a local ethic committee (Société de Réanimation de Langue Française, CE SRLF 19-04). According to French law, need for informed consent was waived. In accordance with the French legislation, the database was declared to the CNIL (“Commission Nationale de l’Informatique et des Libertés”) (number 2221124). All patients were also included in the multicentric CARTTAS study [27].

### Statistical analysis

Continuous variables are described as median (interquartile range [IQR]) and compared between groups using the non-parametric Wilcoxon rank-sum test. Categorical variables are described as frequency (percentages) and compared between groups using Fisher’s exact test. Mortality was assessed by survival analysis. The primary outcome was survival 1 year after ICU admission.

Independent risk factors for 1-year mortality and 1-year progression free survival (PFS) were identified using a Cox model. Conditional stepwise variable selection was performed with 0.2 as the critical P value for entry into the model and 0.1 as the P value for removal. Interactions and correlations between the explanatory variables, the validity of the proportional hazards assumption, the influence of outliers, and the linearity of the relationship between the log hazard and the covariates were carefully checked.

Kaplan–Meier graphs were plotted to express the probability of death from ICU admission to day 360 and probability of PFS. Comparisons were performed using the log-rank test and median survival and PFS with their 95%CI were derived from these analyses.

The missing data rate was 2.5% overall and 0% for the primary endpoint. Imputation of missing data was not performed.

Statistical analyses were performed with R statistical software, version 4.0.5 (available online at http://www.r-project.org/), using the ‘Survival’ packages. Values of *P* < 0.05 were considered significant.

## Results

Seventy-one patients (42 men and 29 women), were included with a median age of 60 years [37–67.5] (Table 1). The underlying hematological malignancy was mostly DLBCL (*n* = 53, 74.6%) with a high tumor burden (64% had extra-nodal involvement), whereas 17 patients had ALL (24%) or multiple myeloma (*n* = 1, 1.4%). During the study period, 166 patients with DLBCL received CAR-T cells in our hospital, which represents an ICU admission rate of 32% in these patients. 43% (17/39) of ALL and 20% (1 in 5 patients) of myeloma patients receiving CAR-T cell therapy also required ICU transfer (Additional file 1: Figure S1). Time since the diagnosis of the malignancy was 14 [9–27] months before CAR-T cell infusion. Patients underwent three lines [3, 4] of chemotherapy. Ten patients with lymphoma received a prior autologous stem cell transplantation. Five patients with leukemia had undergone allogeneic bone marrow transplantation and 11 had received blinatumomab. Median performance status (PS) was 1 [1, 2]. Only few patients had preexisting non-malignant comorbidities, including 6 patients with hypertension, 3 with diabetes and 2 with chronic kidney disease. Median Charlson Comorbidity Index was 4 [2–5]. Median body mass index (BMI) was 24 kg/m^2^ [22–26.3] (Table 1).

**Table 1:** Characteristics of patients at ICU admission

N (%) or Median [IQR]	Overall (*n* = 71)
Demographics	
Age	60 [37–67.5]
Male gender	42 (59%)
Comorbidities	
Hypertension	10 (14%)
Diabetes	3 (4%)
Chronic kidney disease	2 (3%)
Body mass index (kg/m^2^)	24 [22–26]
ECOG Performance status	
Median PS	1 [1–2]
PS 0	11 (15.5%)
PS 1	32 (45%)
PS 2	22 (31%)
PS 3	6 (8.5%)
Hematological malignancy	
Diffuse large B cell lymphoma	53 (75%)
Lymphoblastic leukemia	17 (24%)
Multiple myeloma	1 (1%)
Time between hematological diagnosis and ICU admission (months)	12 [10–16]
Previous hematological treatments	
Number of treatment lines prior to CAR-T cells	3 [3–4]
Autologous stem cell transplantation	10
Bone marrow transplantation	5
Blinatumomab	11
CAR-T cell therapy	
Autologous CAR-T cells	69
Axicabtagene ciloleucel	33 (46%)
Tisagenlecleucel	31 (44%)
Brexucabtagene autoleucel	4 (5.5%)
bb2121	1 (1.5%)
Allogeneic CAR-T cells (UCART19)	2 (3%)
Time between CAR-T cell infusion and ICU admission (days)	6 [4–7]
Clinical and biological features at ICU admission	
Mean arterial blood pressure (mmHg)	68 [60–81]
Temperature (°C)	39.5 [38.5–40]
Neutropenia	47 (66%)
SOFA score	4 [2–6]

*ICU* intensive care unit, *PS* Performance status, *SOFA* Sepsis-related Organ Failure Assessment

All the patients had received conditioning chemotherapy with cyclophosphamide and fludarabine before CAR-T cell infusion. Autologous CAR-T cells were used in all patients except two patients who received allogenic CAR-T cells. Axicabtagene ciloleucel was administered therapy in 33 (46%) patients, while tisagenlecleucel was given to 31 (44%) patients and brexucabtagene autoleucel to 4 (6%) patients (Table 1).

ICU admission occurred 6 days [4–7] after CAR-T cell infusion (Table 1). Patients were mostly admitted because of hemodynamic failure (*n* = 40, 48%) and SOFA at admission was 4 [2–6]. Nine patients (12.6%) had respiratory failure and nine had neurological disorders at ICU admission. Eleven patients (15%) needed close monitoring: among them, all except one subsequently needed organ support or specific treatment (Table 2). At ICU admission, almost all patients presented with fever (median temperature 39.5 °C [38.5–40], and mean arterial pressure was 68 mmHg [60–80].

**Table 2:** CAR-T cell-related complications and management in the ICU

*N* (%) or Median [IQR]	Overall (*n* = 71)
Isolated cytokine release syndrome	33 (46%)
Grade 1	10
Grade 2	17
Grade 3	6
Time between CRS and ICU admission (days)	2 [1–3]
Neurotoxicity	26 (37%)
Grade 1	4/26 (15%)
Grade 2	5/26 (19%)
Grade 3	3/26 (11%)
Grade 4	14/26 (54%)
Time between ICU admission and worst neurotoxicity grade (days)	1 [0–1]
Documented bacterial infection	21 (30%)
Site of infection	
Catheter-related infection	15/21 (71%)
Digestive/biliary tract	4/21 (19%)
Urinary tract	1/21 (5%)
Unknown	1/21 (5%)
Bacteria	
Coagulase negative Staphylococcus	13
Enterobacteriaceae	3
* Pseudomonas aeruginosa*	1
* Enterococcus faecium*	1
* Clostridium difficile*	2
* Paracoccus yeei*	1
Specific treatments	
Tocilizumab	49 (69%)
Median dosage (mg)	800 [560–1480]
Median number of injections	1 [1–2]
Time between ICU admission and tocilizumab (hours)	5 [2–15]
Steroids	40 (56%)
Time between ICU admission and steroids (hours)	23 [5–34]
Siltuximab	9 (12.6%)
Anakinra	2 (2.8%)
Non-specific treatments in the ICU	
Fluid resuscitation at day 1 (mL)	500 [0–1750]
Broad spectrum antibiotics	70 (98%)
Vasopressors	20 (28%)
Mechanical ventilation	4 (6%)
Renal replacement therapy	1 (1.5%)
Outcome	
Death in the ICU	1 (1.5%)
Death in the hospital	8 (12%)
Death at last follow-up	26 (37%)
Median follow-up (months)	6 [2–15]

*CRS* cytokine release syndrome, *ICU* intensive care unit

Characteristics of patients according to the reason for ICU admission are reported in Table 3. Isolated CRS was the most common CAR-T cell-related complication (*n* = 33, 46%), occurring 2 days [1–4] after CAR-T cell infusion (Fig. 1, Table 2). ICU admission was required within a median time of 2 days [1–3] after the onset of CRS. Median grade of CRS was 2 [1, 2], and median duration of CRS, defined by the resolution of symptoms and of fever, was 5 days [3–6]. Only one patient died because of refractory CRS. Twenty-one patients (30%) had documented bacterial infection, mostly from catheter-related infection (71% of the cases) (Fig. 1, Table 2). Patients with catheter-related infection (*n* = 15) had a median number of 2 [2, 3] positive central blood cultures and 0 [0–1] concomitant positive peripheral blood culture. When the culture of the catheter was available, it came back positive in 27% of the cases (3/11). Otherwise, the source of sepsis was attributed to digestive or biliary tracts in four patients, whereas urinary tract infection and bacteremia from unknown origin affected one patient each. In patients with catheter-related infection, coagulase-negative Staphylococcus was usually the causative pathogen (*n* = 13/15) (Table 2).

**Table 3:** Characteristics of CAR-T cells patients according to the reason for ICU admission

Characteristic	CRS *n* = 33	CRS or sepsis *n* = 13	Disease progression *n* = 4	Sepsis *n* = 21	p
Demographics					
Age	56 [27–69]	60 [48–67]	60 [48–64]	65 [44–67]	0.8
Male gender	16 (48.5%)	10 (77%)	4 (100%)	12 (57%)	0.11
Body mass index (kg/m^2^)					
Performance status	1 [1–1]	1 [1–2]	2 [2–2]	2 [1–2]	0.04
Hematological malignancy					0.84
- Diffuse large B cell lymphoma	24 (73%)	10 (77%)	4 (100%)	15 (71%)	
- Lymphoblastic leukemia	8 (24%)	3 (23%)	0	6 (29%)	
- Multiple myeloma	1 (3%)	0	0	0	
Median number of previous treatment lines	3 [2–4]	4 [4–5]	4 [3–5]	4 [3–4]	0.01
Delay between CAR-T cell and ICU admission (days)	6 [4–7]	6 [4–8]	0.5 [− 0.25 to 1]	6 [5–8]	0.01
Clinical features at ICU admission					
Mean arterial blood pressure (mmHg)	69 [60–80]	62 [59–77]	79 [72–86]	66 [62–73]	0.45
Temperature (°C)	39.3 [38.7–39.9]	40.2 [39.4–40.5]	37.5 [36.7–38.2]	39.8 [38.8–40]	0.006
Biological features at ICU admission					
Leucocytes (× 10^9^/L)	0.66 [0.2–1.07]	0.54 [0.1–7.06]	0.58 [0.24–1.11]	0.34 [0.1–1.28]	0.81
Hemoglobin (g/dL)	9.1 [8.6–9.5]	8.6 [8.4–9.2]	8.9 [8.8–9.1]	9 [8.1–9.2]	0.57
Platelets (× 10^9^/L)	72 [48–151]	90 [47–149]	88 [70–95]	78 [18–124]	0.74
Fibrinogen (g/L)	5.1 [4.3–5.8]	6 [4.7–6.9]	4.7 [4.6–5.4]	5.6 [4.9–6.4]	0.54
Creatinin (µmol/L)	61 [52–83]	69 [54–121]	78 [43–117]	57 [48–90]	0.63
Bilirubin (µmol/L)	8.7 [5.6–12.1]	7.6 [6.1–9.9]	14 [9.7–21.1]	6.1 [5–15.5]	0.56
Ferritin (µmol/L)	943 [514–1820]	1184 [643–4238]	7692 [6603–8782]	2434 [912–6114]	0.09
LDH (UI/L)	429 [322–654]	421 [296–507]	611 [525–955]	363 [248–617]	0.37
Lactate (mmol/L)	1.2 [0.8–1.9]	1.7 [1.1–2.7]	1.6 [1.2–1.9]	1.5 [1–2.1]	0.64
SOFA score	4 [2–5]	5 [3–6]	3.5 [2.5–4.5]	4 [2–7]	0.5
Non-specific treatments in the ICU at day 1					
Fluid resuscitation at day 1 (mL)	500 [0–1500]	1500 [0–2000]	0 [0–0]	1000 [0–2000]	0.04
Vasopressors	8 (24%)	4 (31%)	0	6 (29%)	0.64
Mechanical ventilation	1 (3%)	0	0	1 (5%)	0.85
Renal replacement therapy	0	1 (8%)	0	0	0.21
Outcome					
Death in the ICU	0	1 (8%)	0	0	0.21
Death in the hospital	2 (6.1%)	2 (15%)	1 (25%)	3 (16%)	0.53

*ICU* intensive care unit; *SOFA* Sepsis–related Organ Failure Assessment

Thirteen patients (18%) had clinically suspected sepsis or CRS. In these patients, a bacterial infection was mainly suspected. Clinical source of sepsis involved the lung in four patients and the skin and soft tissue in three patients. The remaining four patients (5.6%) with DLBCL experienced disease progression. These last patients presented with less organ failure, ICU admission being required for close monitoring or specific procedures (chest tube insertion, drainage of specific ascitic fluid, high-flow oxygen in relation with specific pulmonary infiltration, monitoring of acute cardiac insufficiency).

ICANS was reported in 26 (37%) patients and was often severe with a worst grade of 4 [2–4] according to the CARTOX grading system (median score was 2 [0–3]), achieved 1 day [0–1] after ICU admission and 5 days [4–7] after CAR-T cell infusion (Table 2). In this cohort, neurotoxicity was never isolated, all patients with ICANS presented with concomitant features of CRS or sepsis. Other organ toxicities were often mild and included acute kidney injury (median grade of 1 [1]) and liver cytolysis (grade 1 [1, 2]) in 21 (30%) patients each [29]. Ten patients (14%) also experienced coagulation disorders, which resulted in a decreased fibrinogen level. This latter complication was often delayed, occurring within 8.5 [7–10] days after CAR-T cell infusion.

Within one day of ICU admission, patients received 500 mL [0–1750] of fluid resuscitation, vasopressors (*n* = 18, 25%) and broad-spectrum antibiotics (98%). Two patients required mechanical ventilation at day 1, and two additional patients were intubated during ICU stay (*n* = 4, 5.6%) (Table 2). Regarding specific treatments, tocilizumab had been given to 49 (69%) patients within 5 h [2–15] after ICU admission, and 40 patients (56%) received corticosteroids. Thirty patients (42%) received both treatments. Dexamethasone was most commonly used drug (in 80% of the patients), 23 h [5–34] after ICU admission, at a median initial dose of 40 mg per day [20–40]. Nine patients (12.6%) also received siltuximab, anti-IL 6 monoclonal antibody, as a second-line therapy during CRS (median dose of 800 mg [700–1600]), mainly in accordance with clinical trial protocols. Anakinra, a recombinant IL-1 receptor antagonist (IL-1ra) was administered to two patients (2.8%) presenting with refractory neurotoxicity (Table 2).

ICU length of stay was 4 days [2–6]. ICU mortality rate was 1.4%, while eight patients died in the hospital (11%) (Table 2). Palliative care was decided for 3 patients with disease progression. Characteristics of patients according to the outcome at 1 year are reported in Table 4.

**Table 4:** Characteristics of CAR-T cell patients according to the outcome at 1 year

N (%) or Median (IQR]	1-year survivors (*n* = 45)	1-year decedents (*n* = 26)	*p*
Demographics			
Age	60 [32–68]	58 [41–65]	0∙84
Male gender	24 (53%)	18 (69%)	0∙29
Comorbidities			
Hypertension	4 (9%)	6 (23%)	0.19
Diabetes	3 (7%)	1 (4%)	1
Chronic kidney disease	2 (4.5%)	0	0.73
Body mass index (kg/m^2^)	24.5 [22–27]	23.5 [22–25.5]	0.40
Performance status	1 [1–2]	2 [1–2]	0.023
Hematological malignancy			0.73
Diffuse large B cell lymphoma	33 (73%)	20 (77%)	
Lymphoblastic leukemia	11 (24.5%)	6 (23%)	
Multiple myeloma	1 (2%)	0	
Median number of previous treatment lines	3 [2–4]	4 [3–5]	0.12
Time between CAR-T cell infusion and ICU admission (days)	6 [4–8]	5 [3–7]	0.21
Reason for ICU admission			0.04
CRS	22 (49%)	11 (42%)	
CRS or sepsis	10 (22%)	3 (11.5%)	
Sepsis	13 (29%)	8 (31%)	
Disease progression	0	4 (15.5%)	
Clinical and biological features at ICU admission			
Mean arterial blood pressure (mmHg)	66 [60–78]	70 [63–82]	0.24
Temperature ( °C)	39.9 [38.9–40.3]	38.9 [38–39.7]	0.003
Neutropenia	25 (55.5%)	22 (85%)	0.02
SOFA score	4 [2–6]	5 [2–7]	0.21
Non-specific treatments in the ICU at day 1			
Fluid resuscitation at day 1 (mL)	1000 [0–2000]	500 [0–1500]	0.23
Vasopressors	10 (22%)	8 (31%)	0.61
Mechanical ventilation	2 (4.5%)	0	0.73
Renal replacement therapy	0	1 (4%)	0.78

*CRS* cytokine release syndrome, *ICU* intensive care unit, *SOFA* sepsis-related organ Failure Assessment

**Fig. 2:**
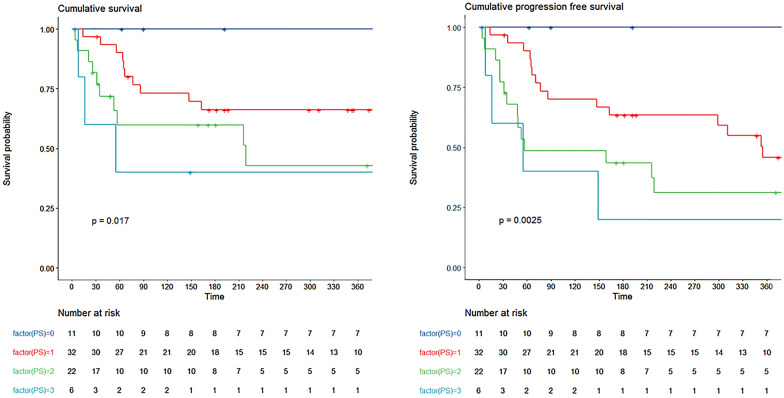
Overall survival (**A**) and progression-free survival (**B**) according to performance status (PS)

**Fig. 3:**
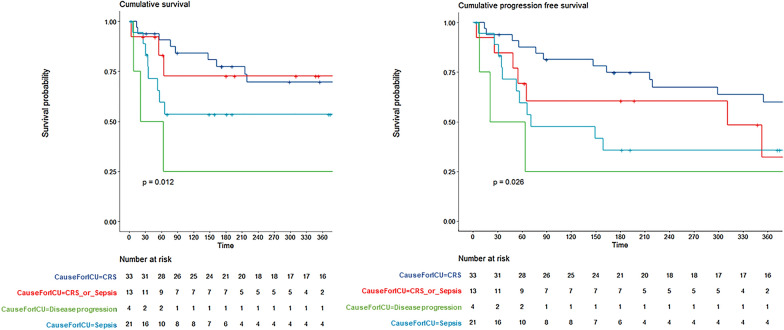
Overall survival (**A**) and progression-free survival (**B**) according to the reason for ICU admission

By multivariable analysis, reason for ICU admission (HR 4.02 disease progression vs. sepsis or CRS [95%CI 1.10–14.65]), PS (HR 1.97 per point [95%CI 1.14–3.41]) and SOFA score (HR 1.16 per point [95%CI 1.01–1.33]) associated with mortality (Figs. 2 and 3, Additional file 1: Table S3).

In the entire cohort, median survival was 17.8 months [95%CI 17.1-NA] and progression-free survival was 11.6 Months [95%CI 5.2-NA]. The median follow-up was 6 months [2–15]. At the last follow-up, 25 patients had a complete response (35%) and 6 (8%) had a partial response; 26 patients (37%) were deceased and 11 (15%) experienced disease progression. The primary cause of death was related to disease progression or relapse, while three patients died from infection (data provided in an online data supplement, Additional file 1: Table S4). Of note, all the patients admitted to the ICU for disease progression died (n = 4).

## Discussion

CAR-T cell therapy is a promising treatment in refractory hematological malignancies. We describe 71 critically ill patients who received CAR-T cells and who experienced severe complications requiring ICU admission.

Bacterial infections are frequent in these immunocompromised patients. Here, we found an incidence of documented bacterial infections of 30%, which is consistent with previous published data. In the literature, the occurrence of any infection is reported in 23% to 42% of adult patients during the 1st month after CAR-T cell infusion [26, 27, 32–34]. Hill et al. described 133 patients receiving CAR-T cell therapy, in whom infections were mainly of bacterial origin (55 to 65% of cases), and more rarely, of viral or fungal origin [26]. While our patients were hospitalized in the ICU 6 days [5–8] after CAR-T cell therapy, the median time to the first infection is reported between 6 and 12 days after CAR-T cell infusion [26, 34]. Interestingly, in our study catheters were often involved, in nearly three in four septic patients, which has never been previously reported. Intensivists should bear in mind that the occurrence of fever in a patient who has received CAR-T cells is likely to be related to bacterial infections. Consequently, in accordance to published clinical practice guidelines, we recommend that these immunocompromised patients should urgently receive broad-spectrum antibiotics [11]. Moreover, as catheter-related infections seem frequent in this particular population, catheter removal should always be considered. Bacterial infections can be severe, even if life-threatening or fatal infections are infrequent after CAR-T cell therapy, death occurring in less than 10% of the cases [26]. In our cohort, we did not find any association between death and ICU admission for sepsis. The majority of catheter-related infections in our cohort can explain this. Indeed, critically ill onco-hematological patients with catheter-related infections are known to have a good prognosis in the ICU, source control being probably a major element of the outcome [35]. Lecronier et al. reported a cohort of 68 immunocompromised patients with catheter-related infections, in whom ICU mortality was very low (9%) [36]. Even if the small number of deaths in our study does not allow strong affirmations, few patients admitted for sepsis died in the hospital (*n* = 3, 14% of septic patients). Our data support that ICU admission should be encouraged in case of sepsis in a patient who has received CAR-T cells, especially if catheter-related infection is suspected, due to the expected favorable short-term outcome.

Our study confirms that CRS and sepsis may be difficult to distinguish for intensivists. We chose a classification that allows the absence of overlap between the different groups of patients, but patients with “sepsis or CRS” share clinical and biological features of CRS with a suspected clinical infection without microbiologically documentation. In fact, studies conducted in neutropenic hematological patients showed that only half of these critically ill patients have a documented bacterial infection. Consequently, we cannot exclude that some patients classified as “sepsis or CRS” had a bacterial sepsis without microbiological confirmation. Hill et al. previously reported that 23% of CAR-T cell recipients experienced any infection during the 1st month after CAR-T cell infusion. In this study, CRS severity was the only factor associated with infection in a multivariable analysis [26]. As no clinical feature is pathognomonic in these immunocompromised patients with fever and organ failure, some authors tried to analyze biomarkers to distinguish these two entities. Diorio et al. have recently identified 23 cytokines that were significantly different between patients with sepsis and CRS: they demonstrated that the combination of IFN gamma and IL1 beta dosages was able to classify subjects as having CRS or sepsis [37]. Nevertheless, these results should be interpreted cautiously as cytokines levels are highly dynamic over time and no patient had received CAR-T cells in the “sepsis” group. Moreover, these laboratory tests are not performed routinely and intensivists cannot rely on the result to make a diagnosis at ICU admission. The most important message is that CRS and sepsis are highly close and antibiotics should always be promptly administered in the ICU, in accordance with current therapeutic recommendations [15].

Early disease progression after CAR-T cell infusion was associated with a poor outcome in our study, as 3 in 4 patients were deceased 3 months later. Indeed, early progression after CAR-T cell infusion is associated with a poor outcome. In a French cohort study of lymphoma patients receiving CAR-T cells, among the patients who failed treatment, 49% of failures occurred during the 1st month after infusion. Median progression-free survival in these patients was 7.4 months. Predictive factors of early progression were extra-nodal involvement (≥2 sites) and lymphoma burden (estimated by the LDH level and total metabolic tumor volume) [38]. The goals of intensive care therapy should be discussed, even ICU admission may be required to exclude differential diagnoses. In particular, inflammation as a result of CAR-T cell expansion should be ruled out by performing radiological exams and biopsy if feasible [39]. Given the poor prognosis of these patients when disease progression is confirmed, invasive procedures in the ICU may be considered as unreasonable obstinacy and palliative care should be considered.

ECOG Performance status (PS) is a well-established prognostic factor in cancer patients. In a cohort of 418 patients with various hematological malignancies (DLBCL, ALL and multiple myeloma), Faucher et al. demonstrated that a poor PS was correlated with a higher day 28-mortality rate [40]. In the setting of ICU patients, large studies evaluated the impact of PS on the outcome, and found that PS impairment was independently associated with hospital mortality [41, 42]. Moreover, in critically ill patients with hematological malignancies, PS was also correlated with survival [43, 44]. Data regarding PS in CAR-T cell patients are scarce. Indeed, in the first clinical trials evaluating the safety and efficacy of CAR-T cell products (JULIET, ZUMA-1, TRANSCEND), PS was part of inclusion criteria and no patient with PS > 1 was enrolled [2–4]. In the real world setting, half of patients who receive CAR-T cell therapy would not have met these eligibility criteria due to PS > 1 [45, 46]. In this study, we report for the first time the strong influence of PS at ICU admission on the outcome in critically ill patients who have received CAR-T cells, although they have few comorbidities due to prior selection. This is consistent with preliminary data reported by Jacobson et al. in a multicentre retrospective study including 76 patients with relapsed or refractory large B-cell lymphoma. In this study, while there was no increased toxicities according to PS, the outcome was worse in patients with altered PS, in relation with a lack of response [47]. As PS was independently associated with mortality in our study, our data support that evaluation of PS is crucial to determine whether ICU admission could be beneficial in these patients.

This study has several limitations. First, due to its single-center design, data regarding specific management of CAR-T cell-related complication may not be generalizable to other expert centers. Particularly, the admission modalities to the ICU may vary across centers and countries, as patients were admitted very early in our center, based on previous studies that showed that delayed admission to the ICU was correlated with a poor prognosis. Nevertheless, we followed the current published guidelines about management of CRS and neurotoxicity. Second, the classification of the patients into four distinct groups was chosen arbitrarily and neurotoxicity is not considered separately, but it allows the absence of overlap between the different groups of patients. In this line the small sample size in the disease progression subgroup translate into wide confidence interval and uncertainty that should be taken into account when interpreting our results. Third, we have chosen to focus only on bacterial infections as regard to their severity and deliberately disregard viral infection which may need to be assessed in a specific study. Of note, no fungal infection was documented during first ICU stay. Last, although overall survival and PFS are encouraging, it must be noted that patients entering CAR-T process are a vastly selected population of patients. Thus, respective input of CAR-T per se when compared to patients’ selection on outcome remains to be determined. Nevertheless, our results suggest a meaningful survival and PFS in this selected group of patients.

## Conclusions

While there are few data about critically ill patients receiving therapy, this study provides interesting information. In these immunocompromised patients, documented bacterial infections are frequent. As they are usually related to catheter infection, removal of the latter should be considered at ICU admission. Performance status strongly influences the outcome: while survival is constant in patients with very good PS, patients with impaired PS and patients admitted in the ICU for disease progression had a poor survival. These two prognostic factors should be assessed to determine whether ICU admission could be beneficial in these patients.

## Supplementary Information

**Additional file1**: **Figure S1**. Patient flow chart. **Table S1**. CRS grading (adapted from Lee et al, Biol Blood Marrow Transplant 2019). **Table S2**. Neurotoxicity grading according to the CAR-T cell therapy-associated TOXicity score (CARTOX) (adapted from Lee et al, Biol Blood Marrow Transplant 2019). **Table S3**. Variables associated with mortality by multivariable logistic regression. **Table S4**. Causes of death

## Data Availability

The data set supporting the conclusions of this article is included within the article (and its additional files).

## Abbreviations

ALL: Acute Lymphoblastic Leukemia
CARTOX: CAR-T cell therapy-associated TOXicity score
CRS: Cytokine Released Syndrome
CTCAE: Common Terminology Criteria for Adverse Events
DLBCL: Diffuse Large B Cell Lymphoma
ECOG: Eastern Cooperative Oncology Group
ICANS: Immune effector Cell-Associated Neurotoxicity Syndrome
ICU: Intensive Care Unit
PFS: Progression Free Survival
PS: Performance Status
SOFA: Sepsis-Related Organ Failure Assessment
