# Supplementary material for: The use of ICU resources in CAR-T cell recipients: a hospital-wide study
Source: Ann Intensive Care. 2022 Aug 17;12:75. doi: 10.1186/s13613-022-01036-2 (PMC9385897; doi:10.1186/s13613-022-01036-2)
Supplement: Supplementary file 1 — Additional file1: Figure S1. Patient flow chart. Table S1. CRS grading (adapted from Lee et al, Biol Blood Marrow Transplant 2019). Table S2. Neurotoxicity grading according to the CAR-T cell therapy-associated TOXicity score (CARTOX) (adapted from Lee et al, Biol Blood Marrow Transplant 2019). Table S3. Variables associated with mortality by multivariable logistic regression. Table S4. Causes of death [file 13613_2022_1036_MOESM1_ESM.docx]

**SUPPLEMENTARY MATERIAL**

**Figure S1: Patient flow chart**

**
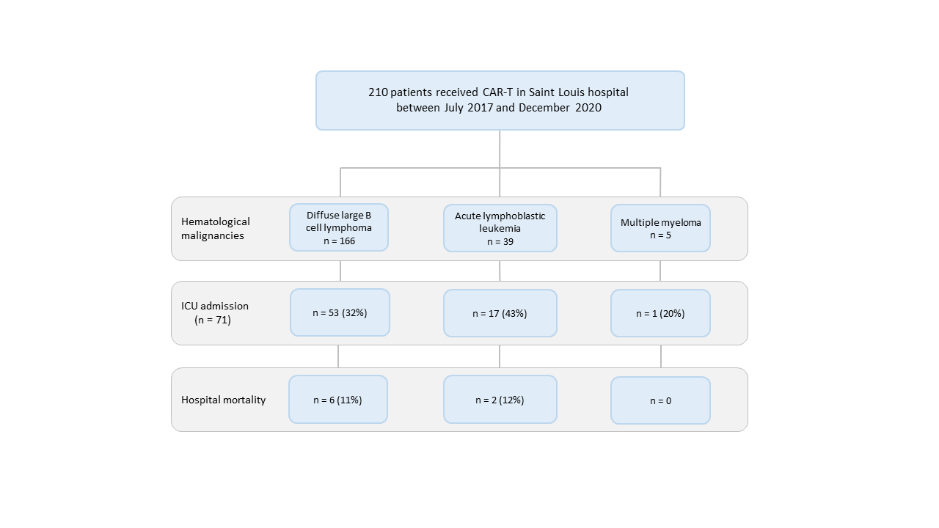
**

**
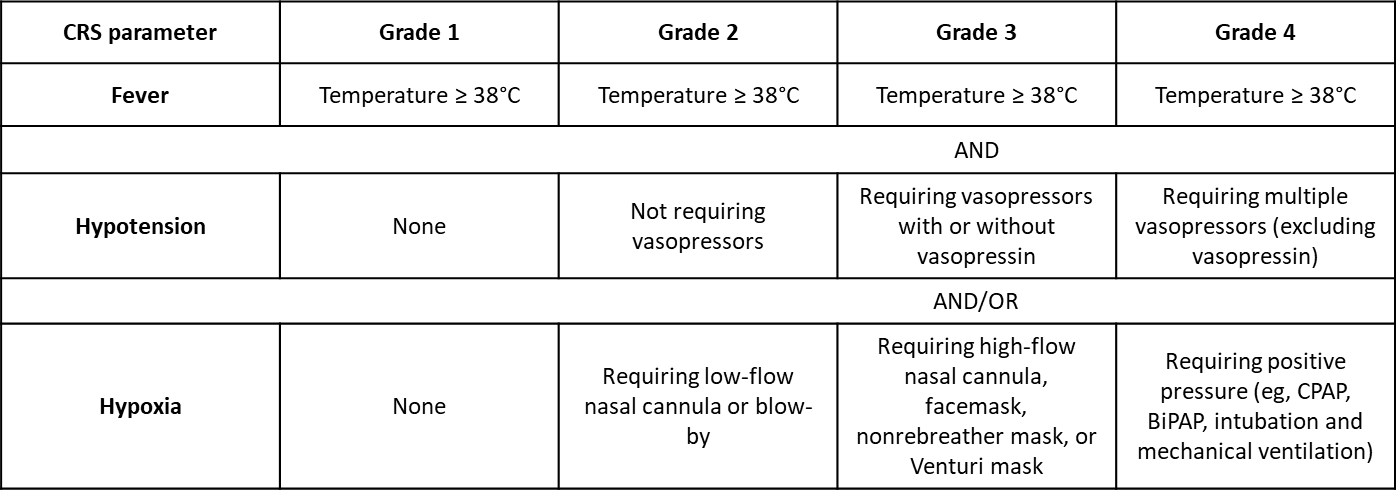
Table S1. CRS grading** (adapted from Lee et al, Biol Blood Marrow Transplant 2019)

**Table S2. Neurotoxicity grading according to the** **CAR-T cell therapy-associated TOXicity score (CARTOX)** (adapted from Lee et al, Biol Blood Marrow Transplant 2019)


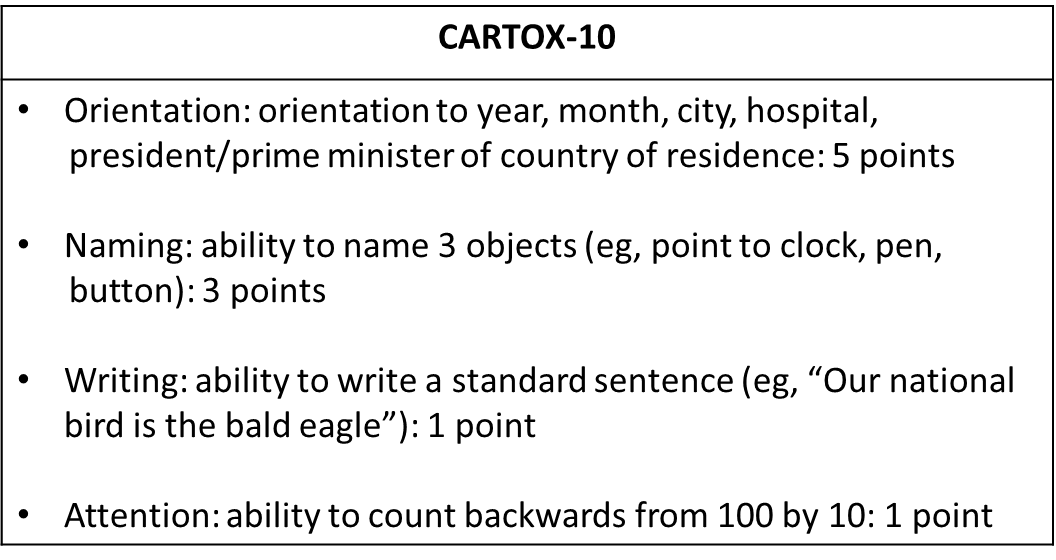

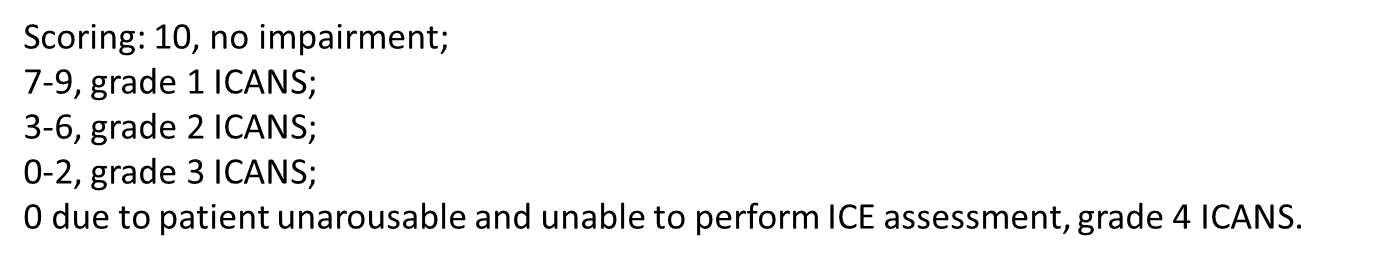


**Table S3.** **Variables associated with mortality by multivariable logistic regression**

| **Variables** | **Odds ratio** | **95% confidence interval** | ***P* value** |
| --- | --- | --- | --- |
| **Performance status** | 1.97 | (1.14-3.41) | 0.015 |
| **SOFA score** | 1.16 | (1.01-1.33) | 0.031 |
| **“Disease progression”** | 4.02 | (1.10-14.65) | 0.035 |
| **“Sepsis”** | 1.81 | (0.68-4.86) | 0.236 |
| **“Sepsis or CRS”** | 0.62 | (0.17-2.29) | 0.473 |

**Table S4.** Causes of death

| **Characteristic** | **Overall**  **(n = 26)** | | **≤ day 30**  **(n = 7)** | **≤ day 90**  **(n = 17)** | **≤ day 180**  **(n = 21)** | **> day 180**  **(n = 5)** |
| --- | --- | --- | --- | --- | --- | --- |
| **Refractory CRS**  **Disease progression/relapse**  **Infection**  **ARDS**  **Refractory status epilepticus** | | 1 (4%)  20 (77%)  3 (11%)  1 (4%)  1 (4%) | 1  4  2  0  0 | 1  13  3  0  0 | 1  17  3  0  0 | 0  3  0  1  1 |

CRS, Cytokine Release Syndrome; ARDS, Acute Respiratory Distress Syndrome
